# A PCR-independent approach for mtDNA enrichment and next-generation sequencing: comprehensive evaluation and clinical application

**DOI:** 10.1186/s12967-024-05213-8

**Published:** 2024-04-25

**Authors:** Dong Liang, Lin Zhu, Yuqing Zhu, Mingtao Huang, Ying Lin, Hang Li, Ping Hu, Jun Zhang, Bin Shen, Zhengfeng Xu

**Affiliations:** 1 Department of Prenatal Diagnosis, Women’s Hospital of Nanjing Medical University, Nanjing Women and Children’s Healthcare Hospital, Nanjing, 210004 China; 2 State Key Laboratory of Reproductive Medicine and Offspring Health, Women’s Hospital of Nanjing Medical University, Nanjing Women and Children’s Healthcare Hospital, Nanjing Medical University, Nanjing, 211166 China

**Keywords:** PCR-independent mtDNA enrichment, mtDNA sequencing, NGS, Methods comparison, Prenatal screening

## Abstract

**Background:**

Sequencing the mitochondrial genome has been increasingly important for the investigation of primary mitochondrial diseases (PMD) and mitochondrial genetics. To overcome the limitations originating from PCR-based mtDNA enrichment, we set out to develop and evaluate a PCR-independent approach in this study, named Pime-Seq (PCR-independent mtDNA enrichment and next generation Sequencing).

**Results:**

By using the optimized mtDNA enrichment procedure, the mtDNA reads ratio reached 88.0 ± 7.9% in the sequencing library when applied on human PBMC samples. We found the variants called by Pime-Seq were highly consistent among technical repeats. To evaluate the accuracy and reliability of this method, we compared Pime-Seq with lrPCR based NGS by performing both methods simultaneously on 45 samples, yielding 1677 concordant variants, as well as 146 discordant variants with low-level heteroplasmic fraction, in which Pime-Seq showed higher reliability. Furthermore, we applied Pime-Seq on 4 samples of PMD patients retrospectively, and successfully detected all the pathogenic mtDNA variants. In addition, we performed a prospective study on 192 apparently healthy pregnant women during prenatal screening, in which Pime-Seq identified pathogenic mtDNA variants in 4 samples, providing extra information for better health monitoring in these cases.

**Conclusions:**

Pime-Seq can obtain highly enriched mtDNA in a PCR-independent manner for high quality and reliable mtDNA deep-sequencing, which provides us an effective and promising tool for detecting mtDNA variants for both clinical and research purposes.

**Supplementary Information:**

The online version contains supplementary material available at 10.1186/s12967-024-05213-8.

## Introduction

Mitochondria are eukaryotic organelles playing essential roles in a series of cellular processes, including bioenergy production, calcium handling, and intrinsic apoptosis regulation [1, 2]. Each mitochondrion contains multiple copies of their own genome in the mitochondrial matrix, known as mitochondrial DNA (mtDNA). Human mtDNA is a circular double-stranded DNA with 16,569 bp in size, encoding 37 genes, including 13 oxidative phosphorylation (OXPHOS) related polypeptides, 22 tRNAs and 2 rRNAs [3, 4]. Different from the nuclear genome, the mtDNA has a much higher mutation rate due to the exposure to reactive oxygen species and the lack of nucleosome protection. In addition, each human cell contains thousands of mtDNA molecules [5]. As a result, the mtDNA variants often co-exists with the wildtype mtDNA within a cell, known as a state of heteroplasmy [6].

Pathogenic mtDNA variants are potentially related to primary mitochondrial diseases (PMDs). The clinical phenotype of PMDs is highly variable, often affecting the central nervous and musculoskeletal system, leading to severe birth defects and early mortality [7]. In general population, the PMDs caused by pathogenic mtDNA variants present in approximately 1 in 10,000 adults [8]. This is much more prevalent than the PMDs caused by nuclear pathogenic variants, which is in approximately 2.9 per 100,000 adults [9], although more than 1000 mitochondrial proteins are encoded from the nuclear genome [10]. Moreover, most severe pathogenic mtDNA variants tend to be heteroplasmy in nature, where the severity of PMD symptoms correlates with the heteroplasmic fraction (HF) [11, 12]. In this way, it is important to accurately detect mtDNA variants, especially heteroplasmic ones, for both clinical and research purposes.

Currently, there are several major molecular methods for detecting mtDNA variants. Sanger sequencing is often used to reliably detect homoplasmic variants and heteroplasmic variants with high HF [13]. However, this strategy is commonly used to screen known pathogenic variants without providing quantitative information. For purposes of investigating causative rare variants in human diseases or novel mutations on cell biology, whole mtDNA sequencing is required, which is mostly achieved utilizing next generation sequencing (NGS) after PCR-based mtDNA enrichment [14–17]. However, this PCR-based approach can lead to false positive and false negative results of heteroplasmic variants due to mutations introduced by polymerase error and amplification bias [18–24], and the sequencing results can be further distorted when the amplicons lie inside nuclear-mitochondrial sequence (NUMTs) regions. In addition, polymorphisms at primer regions would potentially lead to amplification failure for these mtDNA molecues. Furthermore, due to its relatively long turn-around time, long-range PCR based NGS (lrPCR-NGS) is less preferable for large-scale screening studies. Therefore, an accurate, reliable and effective strategy for whole mitochondrial genome deep-sequencing is urgently needed.

Here, we developed a novel high-throughput mtDNA sequencing method based on mtDNA isolation and Tn5 tagmentation. Our new method can achieve PCR-independent mtDNA enrichment while minimizing the presence of nuclear DNA in the sequencing library, and show its reliability and accuracy for the detection of mtDNA variants, particularly when HF is low. The clinical assessment of this method, conducted on both retrospective and prospective samples, demonstrate the effectiveness of this PCR-independent method in detecting pathogenic variants and heteroplasmic variants with potential biological value, making it a promising approach for prenatal screening and other clinical or research applications in the future.

## Materials and methods

### Sample source

In this study, we utilized 1 mL peripheral blood sample for mtDNA sequencing, which were obtained from leftover blood samples of involved pregnant woman who underwent prenatal cell-free DNA screening at the Department of Prenatal Diagnosis in Nanjing Maternity and Child Health Care Hospital. For the retrospective study, peripheral blood sample was obtained from 4 patients who were previously diagnosed with mitochondrial diseases.

### mtDNA isolation and sequencing library construction

Peripheral blood mononuclear cells (PBMC) were separated from 1 mL fresh peripheral blood samples (within 8 h of blood draw) using Lymphoprep Lymphocyte Isolation Solution (Stemcell, Canada) and SepMate Centrifuge Tube (Stemcell, Canada), and approximately 40,000 fresh PBMC were collected for further steps. Urine derived cells were harvested from 60 mL urine sample by centrifugating at 500 × g for 12 min followed by washing with 0.9% NaCl and centrifugating at 500 × g for 3 min. PBMC or urine derived cells were resuspended in 15 μL pre-chilled resuspension buffer (0.1 mM Tris–HCl pH 7.4, 20 μM NaCl, 9 μM MgCl_2_) containing 1% NP40 (Sigma-Aldrich, Missouri, USA), keeping in 4 °C for 3 min. After centrifugation at 10,000 × g for 5 min at 4 °C, 9.5 μL supernatant was collected, which contains a majority of circular mtDNA and a minority of linear nuclear DNA. Next, 1 μL Exonuclease V, 1.2 μL Adenosine 5'-Triphosphate, 1.2 μL NEBuffer™ 4 (NEB, Nebraska, USA) was added to remove any residual linear nuclear DNA in the supernatant, which was incubated at 37 ℃ for 30 min followed by heat inactivation. At this time, mtDNA isolation and enrichment has been completed. The isolated mtDNA was then added with 12.4 μL 2 × TD buffer (0.4 mM Tris–HCl pH 7.6, 0.1 mM MgCl_2_, 4% DMF) and 0.5 μL TTE Mix V50 Tn5 (Vazyme, China), incubating at 37 ℃ for 30 min to obtain mtDNA fragments through tagmentation. The tagmented mtDNA was then recovered using the DNA Clean & Concentrator kit (ZYMO Research, California, USA) and amplified with index primers to construct sequencing library. We established an optional quality control step by performing PCR assay of amplifying nuclear genes (*18S*, *28S*) and mitochondrial genes (*MT-TK*, *MT-ND5*) to validate the efficacy of mtDNA enrichment and nuclear DNA elimination in the library. The libraries were then purified using DNA Clean Beads (Beckman Coulter, Germany) by 0.5 × /0.3 × double size selection and the quantification was measured by Qubit 3.0 (Thermo Fisher Scientific, Massachusetts, USA). Indexed DNA libraries were pooled and sequenced on Illumina NovaSeq 6000, generating paired-end 150 bp reads at 1G data per sample.

### LrPCR-based mtDNA enrichment and sequencing library construction

Full length mtDNA is split into two overlapping segments, each approximately 8.5 kb in length. The primer pairs used were 5’-GCAAATCTTACCCCGCCTG-3’/5’-AATTAGGCTGTGGGTGGTTG-3’ and 5’-GCCATACTAGTCTTTGCCGC-3’/5’-GGCAGGTCAATTTCACTGG-3’. LrPCR was performed using Phanta Max Super-Fidelity DNA Polymerase (Vazyme, China) and 50 ng of total genome DNA. LrPCR reaction condition was 95 °C for 30 s, 25 cycles of 95 °C for 15 s, 60 °C for 15 s, 72 °C for 8 min with a final extension of 72 °C for 5 min. PCR products were then purified using 1% gel and FastPure Gel DNA Extraction Mini Kit (Vazyme, China), and measured using Qubit3.0 (Thermo Fisher Scientific, Massachusetts, USA). The two segments were then pooled with equal moles and tagmented using Tn5 (5 ng amplicon1, 5 ng amplicon2, 2 μL 5 × TTBL, 1 μL TTE Mix V50), incubating at 55 °C for 10 min. The tagments were retrieved using 1 × DNA Clean Beads (Beckman Coulter, Germany), and sequencing library was then constructed using TruePrep DNA Library Prep Kit V2 for Illumina (Vazyme, China). The libraries were purified using DNA Clean Beads by 0.5 × /0.3 × double size selection and the quantification was assessed by Qubit3.0 (Thermo Fisher Scientific, Massachusetts, USA). Libraries were pooled and sequenced on Illumina NovaSeq 6000.

### Quality control and data analysis

The raw sequencing reads were trimmed using trim_galore (v0.6.6) (https://www.bioinformatics.babraham.ac.uk/projects/trim_galore) with following parameters “–paired -q 20 –phred33 –stringency 4 –length 20 –trim-n”. Reads quality control was performed with FastQC (v0.11.9) (https://www.bioinformatics.babraham.ac.uk/projects/fastqc). All the clean reads were aligned to the source genome (Nuclear, GRCh38 and Mitochondrial, NC_012920.1) (http://ftp.ensembl.org/pub/current_fasta/homo_sapiens/dna) using bowtie2 (v2.4.4) (https://github.com/BenLangmead/bowtie2) with default parameters to generate BAM files. Mitochondrial genome reads and nuclear genome reads extraction were performed by Samtools (version 1.7.0) (https://github.com/samtools/samtools/releases). MT reads percentage refers to the percentage of the reads mapped to the mitochondrial genome to all reads mapped to the source genome. The mtDNA variant calling was performed based on GATK Best Practices for SNP/Indel Variant Calling in Mitochondria (https://github.com/gatk-workflows/gatk4-mitochondria-pipeline), with optimization during the variants calling process by setting the downsampling parameter to "–max-reads-per-alignment-start 100,000". The pathogenic variants were visualized in the Integrative Genomics Viewer (IGV) program with BAM files.

### PCR assay

PCR was used to verify the efficiency of mtDNA enrichment. The specific gene regions in the mitochondrial and nuclear genome were amplified and detected with specific primers (Additional file 1: Table S1) and 2 × Taq Master Mix (Vazyme, China). The total reaction volume was 50 μL, containing 25 μL of 2 × Taq Master Mix, 2 μL of 10 μM forward and reverse primer respectively, and DNA 100 ng, add nuclease-free water to 50 μL. The cycle conditions were as follows: an initial denaturation at 98 °C for 30 s, 35 cycles of 98 °C for 10 s, 63 °C for 30 s, 72 °C for 1 min, and a final extension at 72 °C for 5 min. The results were analyzed by visualizing the PCR product using 2% gel electrophoresis.

### Sanger sequencing

We used 40 μL PCR products, amplified from the region containing candidate mtDNA variants, for Sanger sequencing to verify the pathogenic variants. Sanger sequencing reagents and instruments were provided by Applied Biosystems.

### Pyrosequencing

Prepare PCR products according to pyrosequencing requirements. Biotin labeling is applied to one of the primers of PCR for the subsequent single-chain separation and purification, to ensure that there is no free biotin in the primer. PCR system refers to the instructions of PyroMark PCR Kit (QIAGEN, Germany). The samples were then sequenced on PyroMark Q96 Autoprep (QIAGEN, Germany).

## Results

### The mtDNA-isolation-based procedure can achieve highly enriched mtDNA for NGS

**Fig. 1:**
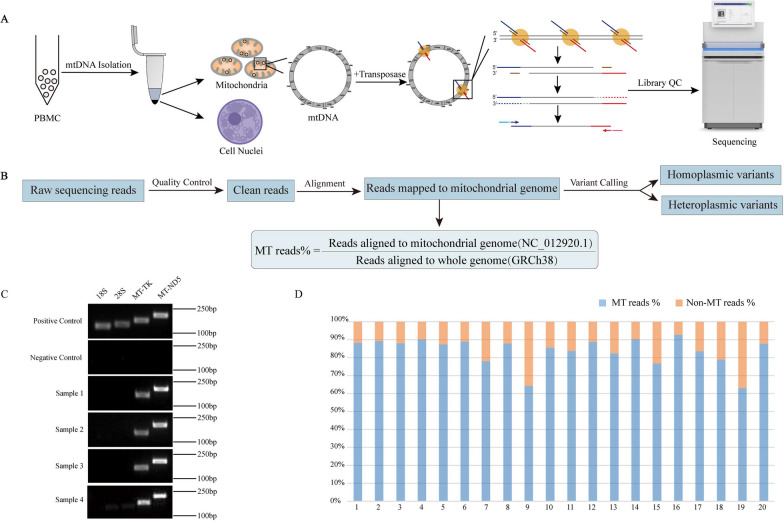
Overview and assessment of Pime-Seq. **A** Workflow of PCR-independent mtDNA enrichment-based sequencing; **B** Flowchart of bioinformatics analysis pipeline for Pime-Seq; **C** Analysis of mtDNA purity after different mtDNA isolation conditions; **D** The MT reads percentage obtained by Pime-Seq on 20 human PBMC samples

To develop and optimize a PCR-independent approach for mtDNA enrichment in human cells, we combined classic cell biology techniques with previously described method for subcellular fractionation to achieve highly efficient separation of mtDNA from nuclear genome [25]. We tested different conditions for mtDNA isolation and enrichment in human PBMC samples, including the use of NP40 and Tween-20 in resuspension buffer and the use of exonuclease after cell lysis. To evaluate the efficacy of mtDNA isolation and enrichment in different conditions, we used PCR assay to amplify and detect nuclear genes (*18S*, *28S*) and mitochondrial genes (*MT-TK*, *MT-ND5*) in enriched DNA (Additional file 8: Fig. S1), which showed an optimal condition involving: (a) the use of resuspension buffer containing 1% NP40 to lyse the cells and isolate mitochondria, and (b) the use of 0.8 unit/μL exonuclease V to degrade the linear nuclear DNA in supernatant. We further performed a PCR assay on the enriched mtDNA from 4 PBMC samples, which revealed the mtDNA was convincingly isolated while the nuclear genome was hardly detectable under the given enrichment conditions (Fig. 1C). The circular mtDNA was then tagmented by Tn5 for sequencing library preparation followed by NGS, which is the entire procedure of Pime-Seq (Fig. 1A). The clean sequencing reads were aligned to the mitochondrial genome reference sequence (rCRS, or NC_012920.1). Finally, we called homoplasmic or heteroplasmic variants by GATK Best Practices for SNP/Indel Variant Calling in Mitochondria. We defined that MT reads percentage refers to the percentage of the reads mapped to the mitochondrial genome to all reads mapped to the whole genome reference sequence (GRCh38, mitochondrial genome + nuclear genome) (Fig. 1B). To evaluate this strategy, we performed our new method on 20 human PBMC samples and analyzed the MT reads percentage to determine the relative amount of mtDNA to nuclear DNA in the mtDNA enriched library. The results showed an average of 88.0% ± 7.9% of MT reads. Consistently, the average sequence depth was as high as 16,538 ± 8,987 × (Fig. 1D, Additional file 2: Table S2). These results demonstrated that our PCR-independent approach can achieve efficient mtDNA enrichment before sequencing on PBMC samples, and we thus named this method as PCR-independent mtDNA enrichment-based Sequencing (Pime-Seq). In addition, we tested this approach on 3 urine samples, which showed an average MT reads ratio of 0.5% in the sequencing results with relatively low sequence depth, indicating the current experimental procedure still needs optimization for sample types other than blood (Additional file 3: Table S3).

### Pime-Seq can yield results with high reproducibility and accuracy

**Fig. 2:**
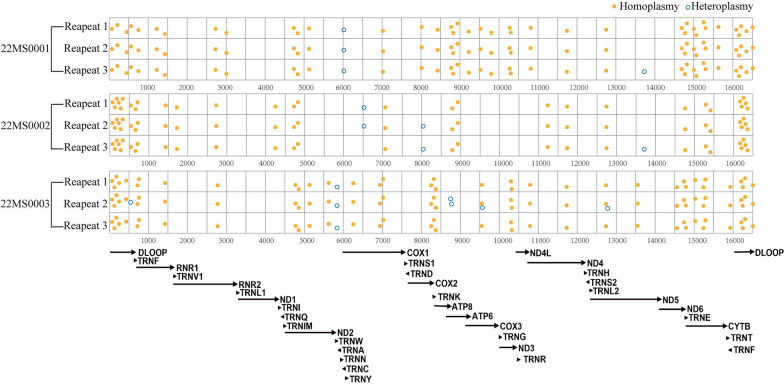
The distribution of all variants identified by Pime-Seq across the mitochondrial genome in 3 technical repeats on 3 human PBMC samples

In order to evaluate the reproducibility of the variant identification using Pime-Seq, we randomly selected 3 human PBMC samples, and performed 3 technical repeats on each sample (Additional file 4: Table S4). The results showed that homoplasmic variants were consistently detected in 3 repeats across all the 3 samples. Similarly, results of variants called with HF greater than 1.4% were consistent in at least two repeats, such as the m.6068C > T in 22TS0001 and the m.5894A > AC in 22TS0003. However, heteroplasmic variants resulting in HF below 1.4% were called without consistency, such as the m. 6510G > A and m.8014A > G variants in 22TS0002 and the m.9576C > A in 22TS0003, indicating potential false positive or false negative results (Fig. 2). These results suggested a detection limit of Pime-Seq for reliable HF, and we therefore set 1.4% as the minimal HF threshold for heteroplasmic variant calling in subsequent studies.

**Fig. 3:**
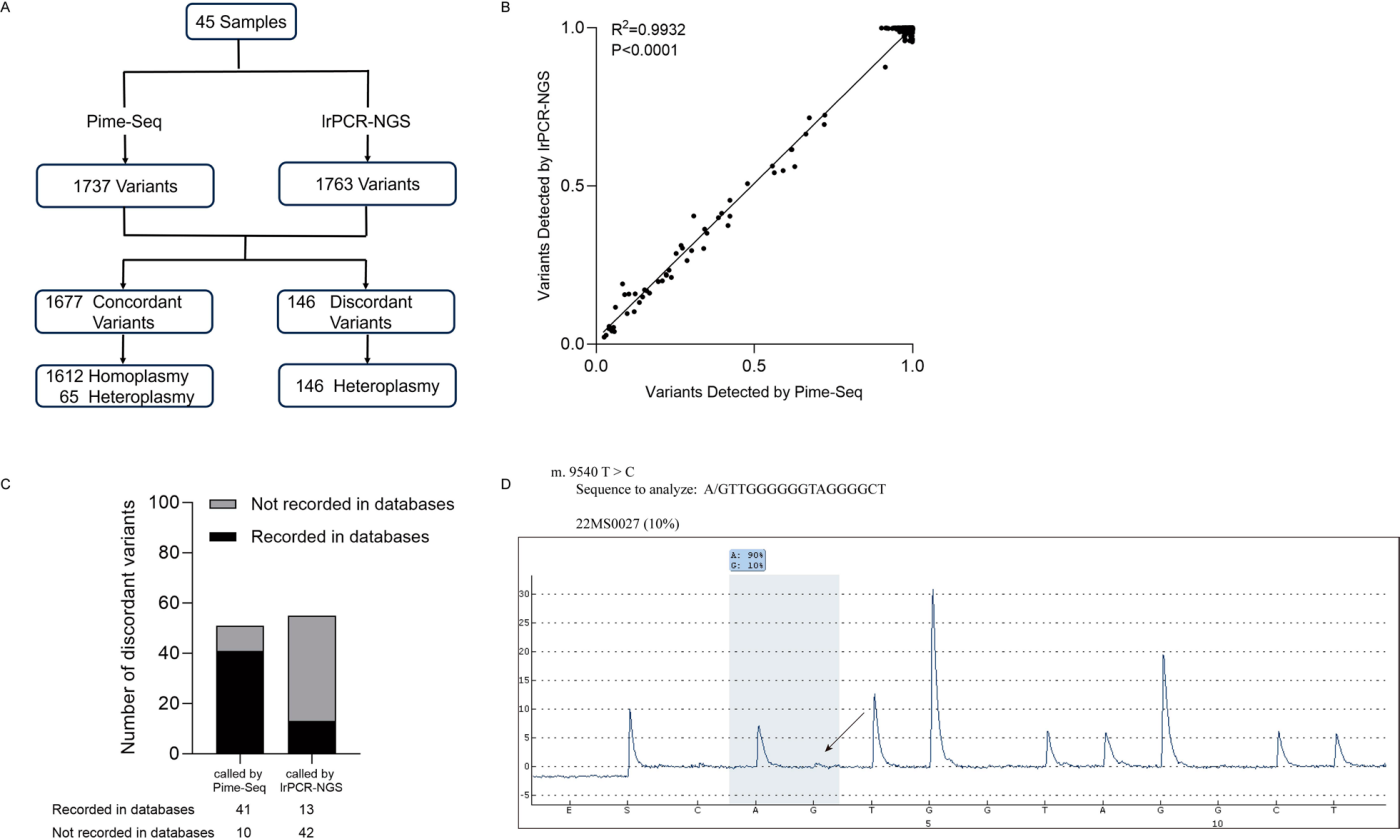
Comparison of Pime-Seq and lrPCR-NGS. **A** flow diagram of the comparison study; **B** HF correlation of the 1677 concordant variants detected by Pime-Seq and lrPCR-based NGS in 45 human PBMC samples. The HF is indicated by the numbers on both the horizontal and vertical axes. Correlations and P values are based on Spearman’s correlation; **C** Evaluating the reliability of the 146 discordant variants by analyzing them in human mitochondrial genome databases; **D** Verification of the discordant result of m.9540T > C in sample 22MS0027 using pyrosequencing

As lrPCR-based NGS is currently used as the most common strategy for mtDNA sequencing, we set out to compare Pime-Seq and lrPCR-NGS in terms of the experimental procedures and the accuracy. We performed both sequencing methods on 45 human PBMC samples in parallel (Additional file 5: Table S5). On average, Pime-Seq took 5 h to complete library construction starting from peripheral blood, whereas lrPCR-NGS required approximately 10 h for the same procedure. From the sequencing results, 1737 variants were called by Pime-Seq and 1763 variants were called by lrPCR-NGS (Fig. 3A). Out of these variants, 1677 were concordant in both methods with high correlation in HF (R^2^ = 0.9932, P < 0.0001, Spearman’s correlation analysis), including 1612 homoplasmic variants and 65 heteroplasmic variants (Fig. 3B). On the other side, a total of 146 discordant variants were only called by one method but not the other in the same sample, which were all heteroplasmic with low HF (Additional file 6: Table S6). A closer analysis of the 146 discrepancies revealed that 80% (41/51) of the putative variants called exclusively by Pime-Seq were found to be recorded in public databases including MitoMap, Human Mitochondrial Genome Database or HelixMTdb, suggesting the detection of these variants by Pime-Seq could reflect a genuine existence. On the other hand, 76% (42/55) of the putative variants called exclusively by lrPCR-NGS were not recorded in any of the three databases, indicating possible false positives in calling these putative variants, probably caused by PCR amplification errors during lrPCR-based enrichment (Fig. 3C). Furthermore, we utilized pyrosequencing to testify the existence of m.9540 T > C in sample 22MS0027, which was called with 4.1% HF by Pime-Seq, but not called by lrPCR-NGS. The pyrosequencing result validated the detection of m.9540 T > C in 22MS0027 (Fig. 3D). Whereas m.9540 T > C was not detected by pyrosequencing in negative control samples (22MS0038 and 22MS0043), in which m.9540 T > C was not called in both Pime-Seq and lrPCR-NGS results (Additional file 9: Fig. S2). These findings demonstrated the accuracy of Pime-Seq results, which is comparable to lrPCR-NGS when calling homoplasmic variants and heteroplasmic variants with high HF, and more reliable when calling heteroplasmic variants with low HF.

### Evaluation of Pime-Seq in a retrospective study

To evaluate its clinical application, we retrospectively conducted Pime-Seq on PBMC samples of 4 previously diagnosed patients carrying pathogenic mtDNA variants, including 1 with homoplasmic m.11778A > G, 2 with homoplasmic m.4300A > G and 1 with heteroplasmic m.8344A > G. The results of Pime-Seq revealed that all the pathogenic variants were accurately detected (Table 1 and Additional file 10: Fig. S3). We further verified all the pathogenic variants using Sanger sequencing, which showed consistent results. It is worth noting that the m.11778A > G variant was recorded as homoplasmic, as well as from our Sanger sequencing result, while the Pime-Seq revealed a heteroplasmic result, with HF at about 94% (Additional file 10: Fig. S3). Above all, our results demonstrated the reliability of Pime-Seq when applied for detecting pathogenic mtDNA variants and PMD genetic diagnosis.

**Table 1:** Retrospective evaluation of Pime-Seq in 4 PMD patients

Sample ID	Previously diagnosed pathogenic variants	Pime-Seq results	
		Variant	HF (%)
22TS0004	Homoplasmic m.11778A > G	m.11778A > G	94
22TS0005	Homoplasmic m.4300A > G	m.4300A > G	99
22TS0006	Homoplasmic m.4300A > G	m.4300A > G	100
22TS0007	Heteroplasmic m.8344A > G	m.8344A > G	65

### Evaluation of Pime-Seq in a prospective study

As a practical strategy for screening pathogenic mtDNA variants in clinical settings are currently lacking, we assessed the application of Pime-Seq in a prenatal screening setting, by utilizing the leftover blood samples from prenatal cell-free DNA screening. A total of 192 healthy pregnant women were randomly selected and included in this study (Table 2). In the 192 prospective samples, Pime-Seq results revealed 7196 variants, including 6743 homoplasmic variants and 453 heteroplasmic variants (Table 3). According to variants with confirmed pathogenic status in Mitomap database, and we identified 4 potential pathogenic variants, including 2 cases of homoplasmic m.1555A > G, 1 case of heteroplasmic m.1555A > G with HF of 2.4%, and 1 case of heteroplasmic m.3243A > G with HF of 13.4% (Table 4). We further verified these variants using Sanger sequencing (Additional file 11: Fig. S4). The homoplasmic m.1555A > G is associated with aminoglycosides-induced hearing loss, and the m.3243 A > G is associated with MELAS and other clinical features when HF is high. As the variant HFs in the 2 cases of heteroplasmic m.1555A > G and heteroplasmic m.3243A > G were relatively low, we therefore only informed the women in the 2 homoplasmic m.1555A > G cases, and planned to add PMD-related questions in the regular postnatal follow-up in all the 4 cases for better health monitoring.

**Table 2:** Clinical information of the 192 apparently healthy pregnant women

Number of subjects (n)	192
Age (years)	
Median	31
Range	20–42
Gestational age (weeks)	
Median	16^+3^
Range	12^+1^–21^+5^
Height (cm)	
Median	162
Range	150–178
Weight (kg)	
Median	58
Range	45–95
BMI (kg/m^2^)	
Median	22.2
Range	17.1–34.4

**Table 3:** Information of the variants called by Pime-Seq in the 192 apparently healthy pregnant women

Total variants	7196
Pathogenic variants (homoplasmy)	2
Pathogenic variants (heteroplasmy)	2
Total homoplasmic variants	6743
Total heteroplasmic variants	453
10% < HF < 95%	76
HF ≤ 10%	377

**Table 4:** Pathogenic mtDNA variants identified by Pime-Seq from the 192 apparently healthy pregnant women

Sample ID	Variant called by Pime-Seq	HF calculated from Pime-Seq (%)	Gene	Mitomap disease description
**22MS0090**	m. 1555A > G	100	MT-RNR1	DEAF; autism spectrum intellectual disability; possibly antiatherosclerotic;
**22MS0122**	m. 1555A > G	98.1	MT-RNR1	DEAF; autism spectrum intellectual disability; possibly antiatherosclerotic;
**22MS0077**	m. 1555A > G	2.4	MT-RNR1	DEAF; autism spectrum intellectual disability; possibly antiatherosclerotic;
**22MS0079**	m. 3243A > G	13.4	MT-TL1	MELAS; Leigh Syndrome; DMDF; MIDD; SNHL; CPEO; MM; FSGS; ASD; Cardiac + multi organ dysfunction

**Fig. 4:**
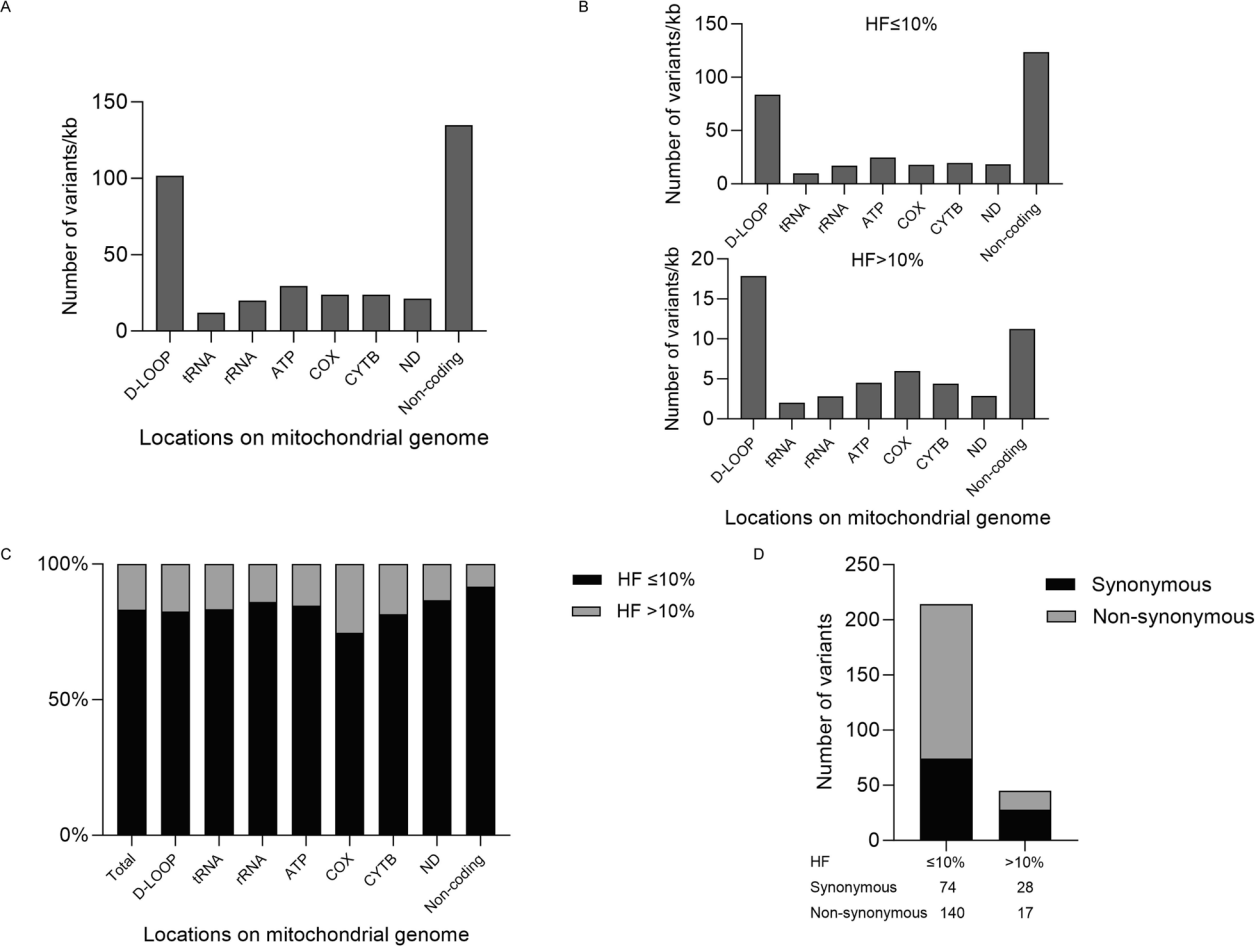
Analysis of the 453 heteroplasmic variants called by Pime-Seq in the 192 prospective samples. **A** Overall distribution of the 453 heteroplasmic variants on the mitochondrial genome; **B** The distribution of the variants with low-level HF and high-level HF; **C** The ratio of variants with low-level HF to high-level HF in different mtDNA locations; **D** The ratio of synonymous variants to non-synonymous in variants with low-level HF and high-level HF

We next characterized the 453 heteroplasmic variants identified by Pime-Seq in these prospective samples, which were classified into high-level HF group (higher than 10%) and low-level HF group (lower than 10%). Most of these heteroplasmic variants were found to localize in the D-LOOP and non-coding region of mtDNA (Fig. 4A), while similar distribution patterns were shown in both high-level HF group and low-level HF group (Fig. 4B), which are both comparable to previous report [26]. Furthermore, the ratio of low-level HF variants in different regions of mitochondrial genome revealed highly consistent, accounting for 74.6% to 91.7% (Fig. 4C). These results also implied the low-level HF variants identified by Pime-Seq are unlikely to be sequencing artifacts, which are expected to distribute the mitochondrial genome uniformly. Interestingly, we found 65.4% (140/214) variants with low-level HF in mtDNA protein gene coding region were non-synonymous variants, while only 37.8% (17/45) variants in high HF group were non-synonymous (Fig. 4D). A possible explanation for this interesting phenomenon is that non-synonymous variants with high-level HF could become extremely detrimental and would be eliminated by selective pressure, suggesting distinct characters with these variants with low-level HF. The detail information of all the 453 heteroplasmic variants can be found in Additional file 7: Table S7. The results from Pime-Seq in this cohort revealed characters of human mtDNA variants in a comprehensive way, which would potentially advance our knowledge of human mitochondrial genome.

## Discussion

Pime-Seq is a novel method for mtDNA deep-sequencing. The key of Pime-Seq strategy is the mtDNA enrichment achieved by mitochondrial isolation, which has revealed satisfactory performance when applied to human PBMC samples. Comparing to other PCR dependent methods, Pime-Seq can achieve a high ratio of mtDNA fragments in the sequencing library through a PCR-independent manner, minimizing the impact of NUMTs contamination, which avoids false positive variants or heteroplasmy shifts introduced by PCR amplification. Additionally, our PCR-independent mtDNA enrichment ensures high sequencing depth of the mitochondrial genome at a relatively low cost and short turn-around time, making it beneficial for large-scale screening or population-wide studies. Similarly, several recent studies have attempted to develop a PCR-independent mtDNA sequencing method to avoid amplification errors and bias introduced by PCR pre-amplification. Legati et al. utilized Mitochondrial DNA Isolation Kit (Abcam, Cambridge, UK) for mtDNA enrichment in their deep-sequencing protocol, resulting in MT reads percentage ranging from 0.3% to 6.8% [27]. Another study by Keraite et al. reported the use of RNA-guided DNA endonuclease Cas9 to specifically enrich the linearized mitochondrial genome for sequencing on long-read sequencing platforms, achieving an average of 54% full-length mtDNA reads when applying on 4 cell lines (HEK293, A549, Capan-2, SH-SY5Y) [28]. Walsh et al. described their PCR-free method to yield purified mtDNA for NGS using differential centrifugation and alkaline lysis, which achieved an average of 75 ± 20% MT reads on fresh mouse tissue. However, this method has not been tested using human cells, and the authors mentioned they were not able to yield adequate mtDNA enrichment using commercial mtDNA enrichment methods on human cells [29]. In comparison, our results showed that Pime-Seq can achieve an average of 88.0% MT reads when applied to human PBMC samples, demonstrating its potential for accurate mtDNA sequencing on human samples with highly enriched mtDNA libraries.

Currently, prenatal screening mainly focused in detecting fetal common aneuploidies, including trisomy 21, trisomy 18, and trisomy 13. Prenatal cell-free DNA screening, one of the most successful clinical applications of NGS, has an excellent performance for this purpose and is broadly adopted worldwide [30]. However, prenatal screening strategy targeting severe genetic disease types other than aneuploidies are limited. In our study, we evaluated the clinical incorporation of Pime-Seq into prenatal screening using a cohort of 192 pregnant women, which revealed a detection rate of 2% (4/192) for pathogenic mtDNA variants. Due to the fact these pathogenic variants would be passed to the fetuses, possibly with increased heteroplasmic level [31, 32], it would be useful for the couple to obtain this information for appropriate genetic counseling and corresponding health monitoring. Furthermore, Pime-Seq and prenatal cell-free DNA screening used PBMC and plasma respectively, which allows one blood draw for both tests. In addition, as the library preparation of Pime-Seq could take as short as 5 h, the total turn-around time could be managed within 7 days, which is similar or shorter than that of prenatal cell-free DNA screening. In this way, our initial assessment revealed the incorporation of Pime-Seq in prenatal screening for pathogenic mtDNA variants would act as an important supplement for current prenatal screening strategy.

It is important to detect heteroplasmic mitochondrial variants with a high sensitivity and accuracy for PMDs, including clinical diagnosis, genetic causes, prevalence, and mtDNA editing evaluation [33–36]. In addition, detection of the heteroplasmic variants can contribute in cancer research [37], population genetics [38], and forensic genetics [39]. Currently, the commercially available lrPCR-NGS is widely used for mtDNA sequencing, but the detection efficacy for variants with low-level HF is limited, and variants with HF lower than a threshold, typically 5% to 10%, are often overlooked due to substantial false-positive calls [26, 40–42]. As Pime-Seq based on PCR independent mtDNA enrichment, it can theoretically achieve an advanced performance when calling heteroplasmic variants with low-level HF, which is also supported by our comparison data. In addition, our initial results showed that variants with low-level HF may have unique characters, such as high ratio of non-synonymous variants. These previously overlooked variants may not be able to accumulate to high-level HF due to their harmfulness to critical respiratory chain proteins and cell survival, and investigation into these variants could potentially lead to discover new variants with extreme pathogenicity with lower HF threshold. Therefore, the reliable detection of variants with low-level HF may provide an informative research angle for mtDNA studies.

It is widely accepted that the mitochondria evolved from α-proteobacterium to form endosymbiosis in the eukaryote history. During this prokaryote-to-eukaryote transition process, the gene-transfer from organelles to the nucleus took place progressively to shape the mitochondrial genome and the nuclear genome, leading to the creation of a number of nuclear copies of pseudo mitochondrial genes, NUMTs [43]. Over 500 NUMTs have been recorded in human reference genome [44], ranging from 39 bp to the entire mitochondrial genome [45], and are found to have population polymorphism and variation among human siblings [46, 47]. These features of NUMTs can lead to serious challenges in identification of pathogenic mtDNA variants [48, 49]. Multiple studies provided evidence that the presence of NUMTs can lead to mis-reported mitochondrial variants when using probe hybridization or PCR amplification to enrich the mtDNA [50–52]. It is worth noting that there have been some interesting reports of paternal mtDNA inheritance [53], but this phenomenon has yet to be confirmed as it may be due to ‘autosomal dominant-like inheritance mode’ actually derived from nuclear elements of mtDNA (NUMTs) [54]. In this way, Pime-Seq may provide an important platform for accurate mtDNA sequencing, even in the presence of NUMTs.

It is clinically critical to choose proper tissue types for genetic testing during PMD diagnosis and management. According to consensus statement from the Mitochondrial Medicine Society and UK best practice guidelines, blood and/or urine DNA are typically acting as the first sample types for genetic analyses, and invasive samples obtained from a diagnostic tissue biopsy are also preferred and informative [36, 55]. Pime-Seq requires to use alive cell samples to prevent degradation of the nuclear genome, and tissues are theoretically suitable when most cells are alive. In this study, we investigated the performance of Pime-Seq on blood samples, as well as a small number of urine samples. We found Pime-Seq on 40,000 PBMCs, isolated from 400 μL to 1 mL fresh peripheral blood, can achieve reliable results. However, although Pime-Seq was able to generate results from 60 mL urine samples with current procedure, the mtDNA enrichment efficiency was less ideal than that on blood samples, possibly due to the high proportion of dead cells, indicating the need for further optimization with regard to urine samples. In addition, we haven’t tested Pime-Seq on clinically relevant tissues such as muscle biopsy, and the performance of Pime-Seq on these precious samples would be investigated in the future. Furthermore, it is also beneficial to apply Pime-Seq in detecting mtDNA copy number variations (CNV) such as single large-scale deletions, but the reliability of CNV results called by Pime-Seq still needs to be validated using mtDNA CNV positive samples, which would be conducted in our future study.

In conclusion, our study here presents a novel low-cost and concise approach, Pime-Seq, that achieves high sensitivity and specificity in analyzing the whole mitochondrial genome on human PBMC samples. Compared to the traditional methods with PCR-based mtDNA enrichment approach, Pime-Seq showed advanced accuracy, especially in identifying variants with low HF. Our study provides evidence that Pime-Seq has a good potential for clinical applications in detecting pathogenic mtDNA variants, particularly in the field of prenatal screening.

### Supplementary Information

**Additional file 1: Table S1.** Primer information.

**Additional file 2: Table S2.** Information of the sequencing reads of the 20 PBMC samples generated by Pime-Seq.

**Additional file 3: Table S3.** Information of the sequencing reads of the 3 urine samples generated by Pime-Seq.

**Additional file 4: Table S4.** All variants called by Pime-Seq in 3 technical repeats on 3 human PBMC samples.

**Additional file 5: Table S5.** Variants called by Pime-Seq and lrPCR-NGS in 45 samples.

**Additional file 6: Table S6. **A total of 146 discordant variants detected in 45 samples by Pime-Seq and lrPCR-NGS.

**Additional file 7: Table S7. **A total of 453 heteroplasmic variants called in 192 prospective samples.

**Additional file 8: Figure S1.** Examining the nuclear DNA and mtDNA post mtDNA enrichment to yield an optimal enrichment condition.

**Additional file 9: Figure S2.** Pyrosequencing result for m.9540T > C in two negative control samples.

**Additional file 10: Figure S3.** Pime-Seq results and Sanger sequencing verification in the 4 previously diagnosed PMD patients.

**Additional file 11: Figure S4.** Pime-Seq results and Sanger sequencing verification of the 4 pathogenic mtDNA variants identified from the 192 apparently healthy pregnant women.

## Data Availability

The datasets analyzed during the current study are available from the corresponding author on reasonable request.
