# Supplementary figures and images for: A PCR-independent approach for mtDNA enrichment and next-generation sequencing: comprehensive evaluation and clinical application
Source: J Transl Med. 2024 Apr 25;22:386. doi: 10.1186/s12967-024-05213-8 (PMC11044483; doi:10.1186/s12967-024-05213-8)

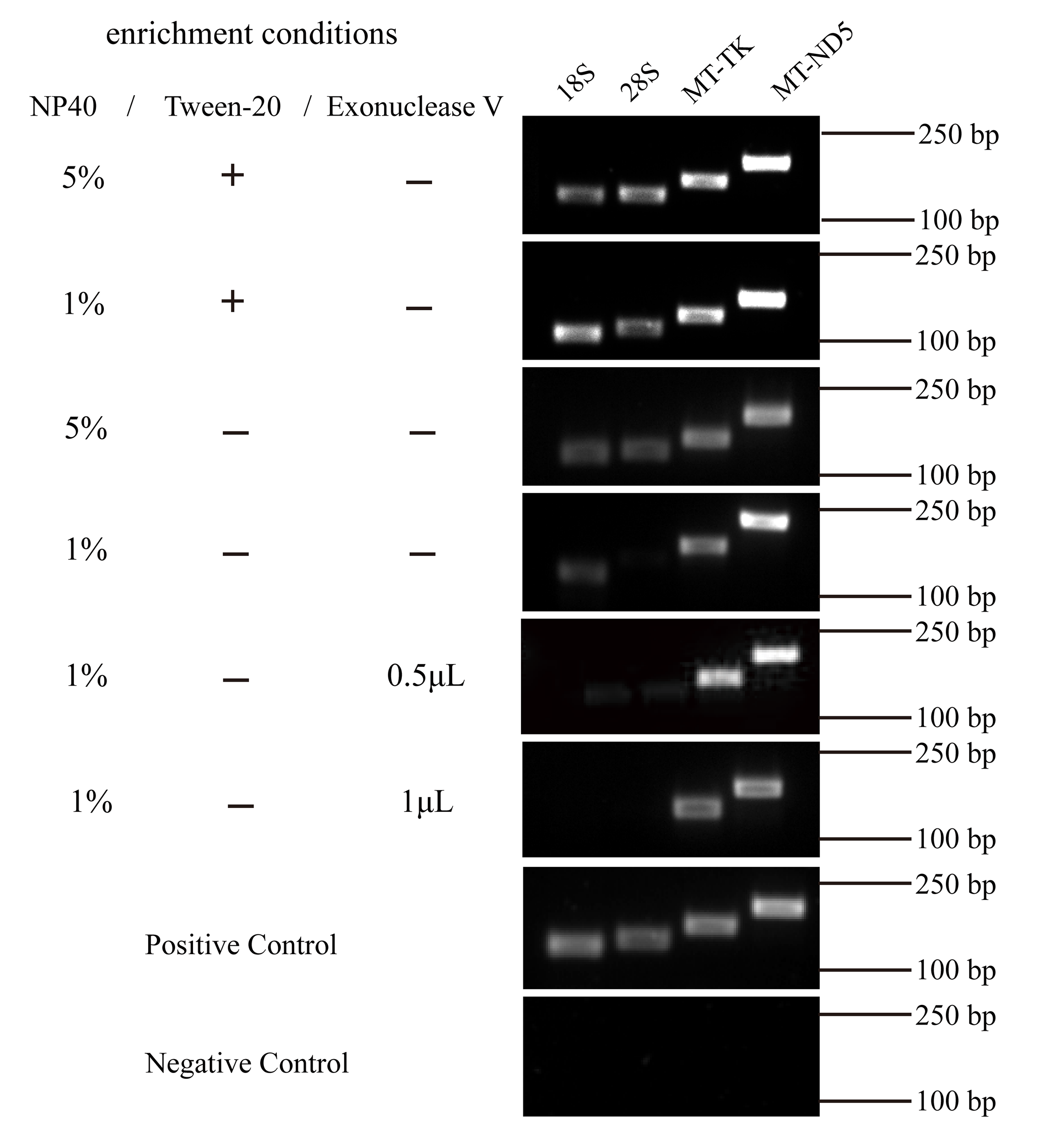

Supplement: Supplementary file 8 — Additional file 8: Figure S1. Examining the nuclear DNA and mtDNA post mtDNA enrichment to yield an optimal enrichment condition. [file 12967_2024_5213_MOESM8_ESM.tif]

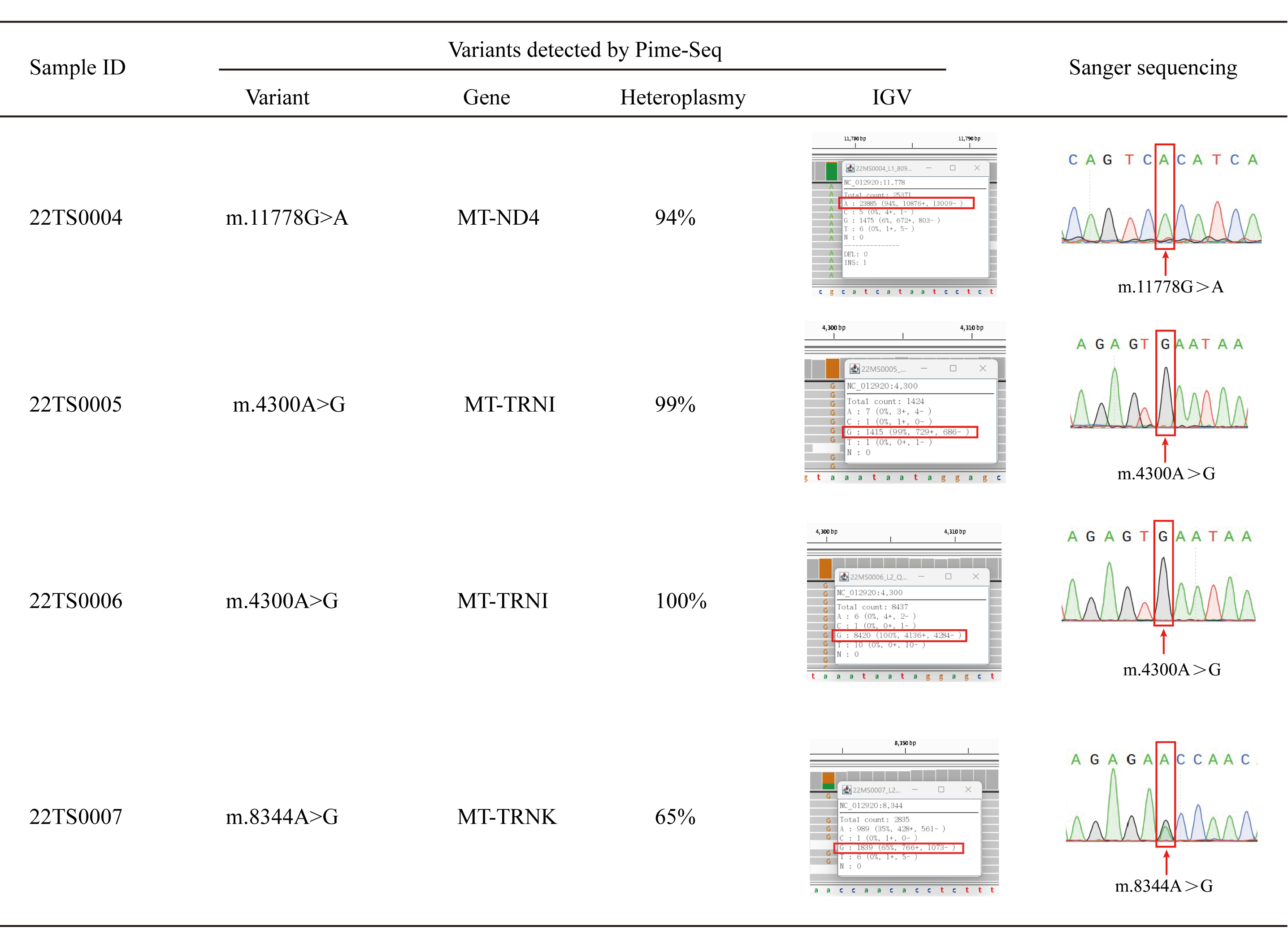

Supplement: Supplementary file 10 — Additional file 10: Figure S3. Pime-Seq results and Sanger sequencing verification in the 4 previously diagnosed PMD patients. [file 12967_2024_5213_MOESM10_ESM.tif]

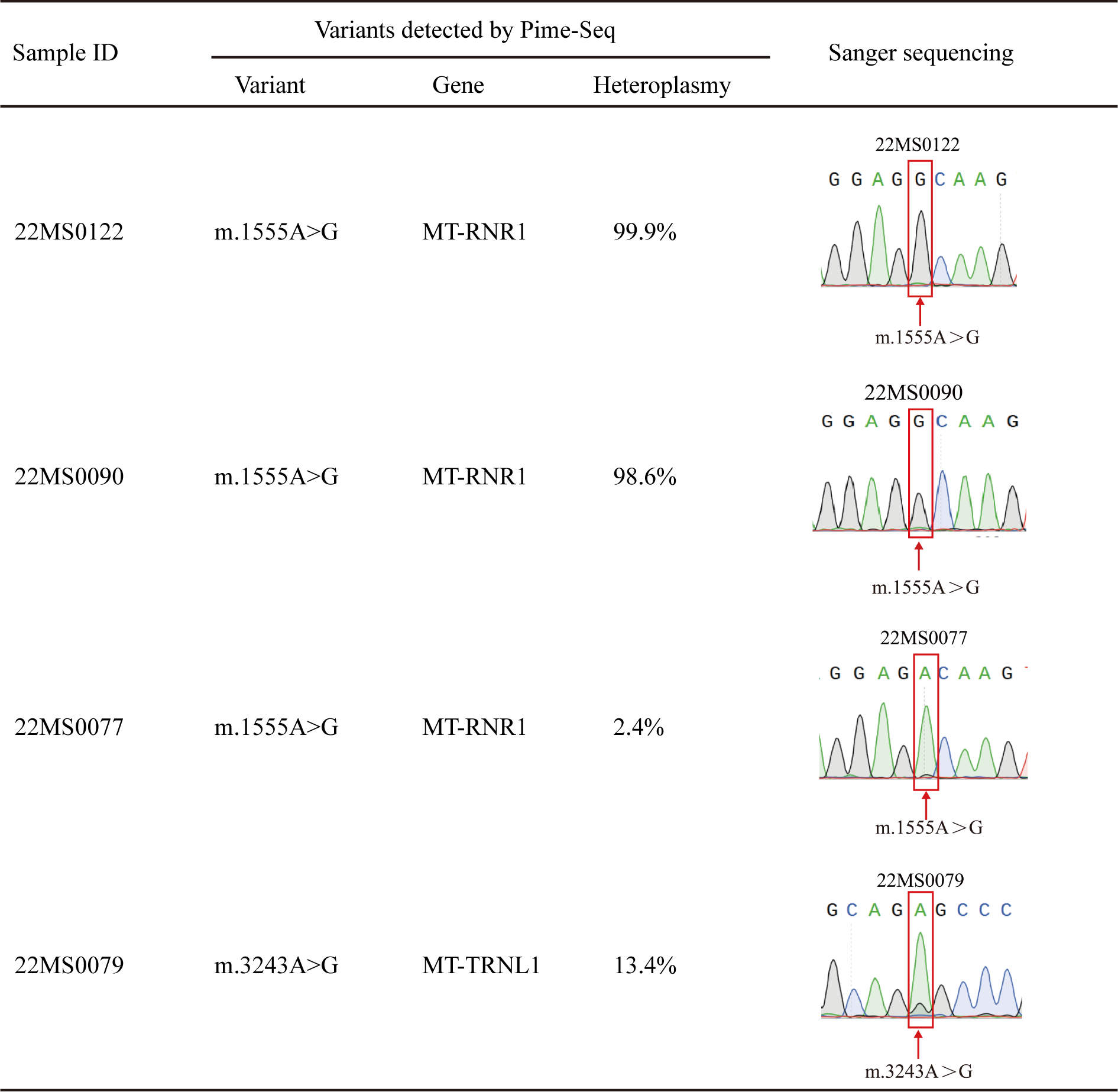

Supplement: Supplementary file 11 — Additional file 11: Figure S4. Pime-Seq results and Sanger sequencing verification of the 4 pathogenic mtDNA variants identified from the 192 apparently healthy pregnant women. [file 12967_2024_5213_MOESM11_ESM.tif]
